# Opposing Actions of Sevoflurane on GABAergic and Glycinergic Synaptic Inhibition in the Spinal Ventral Horn

**DOI:** 10.1371/journal.pone.0060286

**Published:** 2013-04-02

**Authors:** Veit-Simon Eckle, Sabrina Hauser, Berthold Drexler, Bernd Antkowiak, Christian Grasshoff

**Affiliations:** Experimental Anesthesiology Section, Department of Anesthesiology & Intensive Care, Tübingen University Hospital, Eberhard-Karls-University, Tübingen, Germany; University of Arizona, United States of America

## Abstract

**Background:**

The ventral horn is a major substrate in mediating the immobilizing properties of the volatile anesthetic sevoflurane in the spinal cord. In this neuronal network, action potential firing is controlled by GABA_A_ and glycine receptors. Both types of ion channels are sensitive to volatile anesthetics, but their role in mediating anesthetic-induced inhibition of spinal locomotor networks is not fully understood.

**Methodology/Principal Findings:**

To compare the effects of sevoflurane on GABAergic and glycinergic inhibitory postsynaptic currents (IPSCs) whole-cell voltage-clamp recordings from ventral horn interneurons were carried out in organotypic spinal cultures. At concentrations close to MAC (minimum alveolar concentration), decay times of both types of IPSCs were significantly prolonged. However, at 1.5 MAC equivalents, GABAergic IPSCs were decreased in amplitude and reduced in frequency. These effects counteracted the prolongation of the decay time, thereby decreasing the time-averaged GABAergic inhibition. In contrast, amplitudes and frequency of glycinergic IPSCs were not significantly altered by sevoflurane. Furthermore, selective GABA_A_ and glycine receptor antagonists were tested for their potency to reverse sevoflurane-induced inhibition of spontaneous action potential firing in the ventral horn. These experiments confirmed a weak impact of GABA_A_ receptors and a prominent role of glycine receptors at a high sevoflurane concentration.

**Conclusions:**

At high concentrations, sevoflurane mediates neuronal inhibition in the spinal ventral horn primarily via glycine receptors, and less via GABA_A_ receptors. Our results support the hypothesis that the impact of GABA_A_ receptors in mediating the immobilizing properties of volatile anesthetics is less essential in comparison to glycine receptors.

## Introduction

Nociceptive pathways in the spinal cord are activated by surgical interventions like skin incision. In the absence of anesthesia, withdrawal reflexes are triggered by such stimuli. Withdrawal reflexes arise from specific patterns of muscle activation that involve, at the most basic level, left-right and flexor-extensor alternation [1]. These elementary components of locomotor activity are generated by neuronal circuits in the ventral horn of the spinal cord [2]. Thus, it is not surprising that the ventral horn plays a key role in mediating the immobilizing properties of volatile anesthetics [3]. Proper function of movement-generating microcircuits requires precisely timed activation of GABA_A_ and glycine receptors, which are densely expressed in the ventral horn [4]. In the isolated spinal cord, blockade of these receptors abolishes the extensor-flexor alteration of fictive movements [1].

GABA_A_ and glycine receptors belong to the cys-loop superfamily of ligand-gated ion channels [5], [6]. As they are both permeable to chloride ions, activation of these receptors causes inhibition of neuronal excitability in the central nervous system. Within the range of clinically relevant concentrations, volatile anesthetics potentiate the function of GABA_A_ and glycine receptors [7], [8]. Moreover, the molecular structure of the binding site of volatile anesthetics on GABA_A_ and glycine receptors is quite similar [9]. However, it is still controversial to what extent volatile anesthetic-induced inhibition of spinal locomotor networks involves GABA_A_ receptors. On the one hand, an excellent correlation exists between the potency of anesthetic agents to modulate GABA_A_ receptors and their potency to produce immobility *in vivo* [10]. On the other hand, Eger and colleagues postulated that predominantly glycine but not GABA_A_ receptors mediate the immobilizing properties of volatile anesthetics [11], [12]. This hypothesis is based on the observation that GABA_A_ receptor antagonists have a limited capacity to reverse the immobilizing effect of volatile anesthetics.

To further elucidate this discrepancy, we monitored spontaneous GABA_A_ and glycine receptor-mediated inhibitory postsynaptic currents (IPSCs) in ventral horn interneurons. The effects of sevoflurane on glycine and GABA_A_ receptor-mediated inhibition were compared. At 0.5 and 1.5 MAC (minimum alveolar concentration) equivalents of sevoflurane, a similar concentration-dependent prolongation of the decay time of GABAergic and glycinergic IPSCs was observed, indicating that GABA_A_ and glycine receptors were almost equally sensitive to sevoflurane. Interestingly, 1.5 MAC sevoflurane caused a significant reduction in amplitude and frequency of GABA_A_ IPSCs, while such an effect could not be detected for glycine receptor-mediated synaptic events. Thus, at high anesthetic concentrations the potentiation of GABA_A_ receptor currents due to a prolongation of decay time was counteracted by a reduction in IPSC frequency and amplitude.

## Results

### Different Actions of Sevoflurane on GABA_A_ and Glycine Receptor-mediated IPSCs

**Figure 1:**
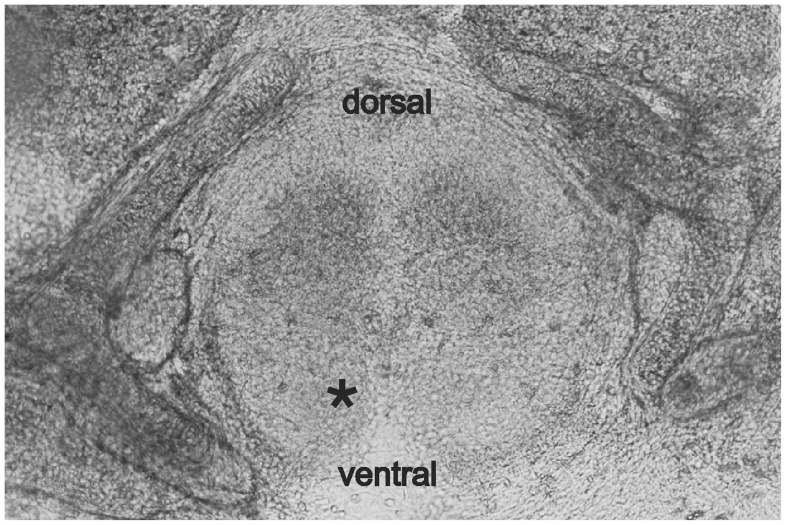
Organotypic spinal cultures. For obtaining organotypic cultures spinal columns were transversely cut and the tissue was cultured (see *Materials and Methods*). These slices are suitable for electrophysiological experiments since interneurons in the ventral horn area are easily accessible, and organotypic synaptic network connections are intact. Ventral horn interneurons were visually identified near the central fissure in lamina VIII (indicated by asterisk). The capacitance of the investigated commissural interneurons was 34.78±3.35 pF (n = 19).

Ventral horn interneurons express GABA_A_ and glycine receptors [4]. Although both types of receptors are sensitive to volatile anesthetics, their impact in spinal inhibition is controversially discussed [11]–[14]. To tackle this issue, GABA_A_ and glycine receptor-dependent synaptic currents were monitored from voltage-clamped commissural interneurons, located in lamina VIII of cultured spinal tissue slices (**Fig. 1** **)**. The effects of sevoflurane on spontaneous inhibitory postsynaptic currents (IPSCs) were studied at two different anesthetic concentrations. At around 0.5 MAC equivalents, volatile anesthetics provide, on the behavioral level, hypnosis, whereas at 1.5 MAC equivalents, motor reflexes are completely blocked [15]. Thus, the effects observed, when investigating sevoflurane concentrations of 0.5 and 1.5 MAC equivalents, are of major interest with regard to the immobilizing properties of this anesthetic. For comparing the actions of sevoflurane on GABA_A_ and glycine receptor-mediated synaptic currents, anesthetic-induced changes in the amplitude, decay time and frequency of spontaneous IPSCs were quantified.

**Figure 2:**
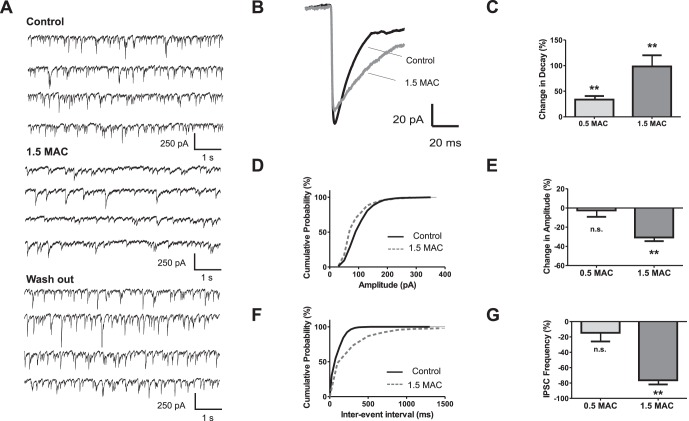
Frequency and amplitude of GABA_A_ IPSCs is significantly reduced by high sevoflurane concentration. (**A**) Sevoflurane effects on GABA_A_ inhibitory postsynaptic currents (IPSCs). Original recording showing control condition (*upper trace*), sevoflurane (1.5 MAC, *middle trace*) and wash out (*lower trace*). GABA_A_ IPSCs were isolated by adding CNQX (50 µM), AP5 (50 µM), and strychnine (1 µM). (**B**) Overlay of averaged IPSCs from the same experiments as depicted in (*A*). Note the prolonged decay time and the slightly reduced amplitude under sevoflurane (1.5 MAC). (**C**) In summary, application of sevoflurane significantly increased the decay times by 33.7±7.1% at 0.5 MAC (n = 8, p<0.01), and 98.4±21.8% at 1.5 MAC sevoflurane (n = 9, p<0.01). Absolute decay time values were 22.3±1.3 ms for control (n = 17), 32.2±2.5 ms for 0.5 MAC (n = 8), and 40.4±5.4 ms for 1.5 MAC (n = 9). (**D**) Cumulative distribution of IPSC amplitudes from the presented experiment. Sevoflurane shifted the population to smaller IPSC amplitudes (*dotted gray line*) compared with values from control condition (*black line*) (**E**) Pooled date showed that amplitudes of GABA_A_ IPSCs remained stable at 0.5 MAC (change in amplitude −2.5±6.7%, n = 8), but were significantly changed by −30.7±3.8% at 1.5 MAC sevoflurane concentration (n = 9, p<0.01). (**F**) Cumulative distribution of inter-event intervals for shown representative traces. Sevoflurane prolonged the inter-event intervals (control *black line*, sevoflurane *dotted gray line*). (**G**) Analysis revealed that the IPSC frequency was essentially reduced at 1.5 MAC sevoflurane (change in frequency by −76.4±5.5% for 1.5 MAC, n = 9, p<0.01), whereas such an impressive effect was not detectable at 0.5 MAC sevoflurane (change in frequency −14.6±11.2%, n = 8). **p<0.01 as indicated.

The decay time of GABAergic synaptic currents was well fitted with a single exponential function. At both tested sevoflurane concentrations the current decays were significantly prolonged (**Fig. 2** **B, C**). In contrast to the decay time constant, the amplitudes of GABAergic IPSCs remained almost unchanged after exposure to 0.5 MAC sevoflurane, but were significantly diminished by 30.7±3.8% at 1.5 MAC equivalents (**Fig. 2** **D, E**). Furthermore, sevoflurane reduced the frequency of spontaneous GABA-dependent IPSCs by 76.4±16.6% at 1.5 MAC sevoflurane equivalents (**Fig. 2** **G**).

The fact that 1.5 MAC sevoflurane decreased both, the amplitude and the frequency of IPSCs, prompted the question, whether these effects were at least in part causally related. This seemed likely because a threshold criterion was used for counting the number of synaptic events (see *Materials and Methods*). We calculated that a 30% decrease of IPSC amplitudes (as produced by 1.5 MAC sevoflurane) reduced the number of events that were detected by our threshold criterion by 19% (see *Materials and Methods*). However, this effect was small as compared to the 76%-drop of IPSC frequency that was observed with the anesthetic. In conclusion, the decrease in frequency of GABA_A_ receptor-mediated synaptic events resulted from two distinct mechanisms. It was, to a smaller part, due to the attenuation of IPSC amplitudes and, to a larger part, produced by a distinct mechanism of anesthetic action.

**Figure 3:**
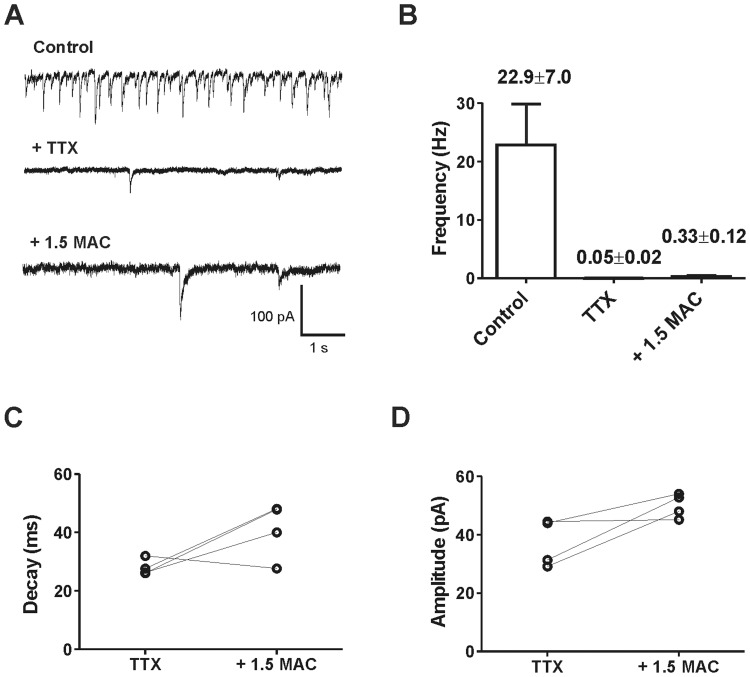
Sevoflurane increased the amplitude and event rate of GABAergic miniature IPSCs. (**A**) Representative recording of spontaneous GABA_A_ receptor-mediated IPSCs (*upper trace*). To focus on postsynaptic sevoflurane effects miniature IPSCs (mIPSCs) were isolated by adding 1 µM tetrodotoxin (TTX, *middle trace*). Subsequently, 1.5 MAC sevoflurane was applied (*lower trace*). (**B**) Pooled data revealed an event rate of 22.9±7.0 Hz for control condition (n = 9). In 5 out of 9 experiments, no single mIPSC could be detected after TTX application. In 4 experiments, the frequency of mIPSC was less than 99% compared to the initial spontaneous event rate without TTX (mIPSC frequency 0.046±0.018 Hz (n = 4). Additional application of 1.5 MAC sevoflurane increased the frequency of mIPSC events to 0.33±0.12 Hz (n = 4). (**C**) Sevoflurane prolonged the mean decay time of mIPSCs (TTX condition 28.0±1.4 ms vs. 40.9±4.8 ms sevoflurane, n = 4, p<0.05). (**D**) Additionally, the mean of mIPSC amplitudes augmented from 37.3±4.1 pA (TTX condition) to 50.0±2.1 pA (n = 4, p<0.05).

In a following step, miniature IPSCs (mIPSCs) were studied subsequently (**Fig. 3**). To completely abolish action potential-triggered synaptic transmission tetrodotoxin (TTX) was applied. After adding TTX to the bath solution, the frequency of GABAergic synaptic events was reduced by about 99%, indicating that the vast majority of spontaneous synaptic events were action potential-dependent (**Fig. 3** **A, B**). Sevoflurane (1.5 MAC) prolonged mIPSC decay times, but increased amplitudes and frequency of mIPSCs (**Fig. 3** **B, C, D**). These findings clearly support the hypothesis that the sevoflurane-mediated decrease in IPSC-frequency and amplitude is action potential-dependent.

**Figure 4:**
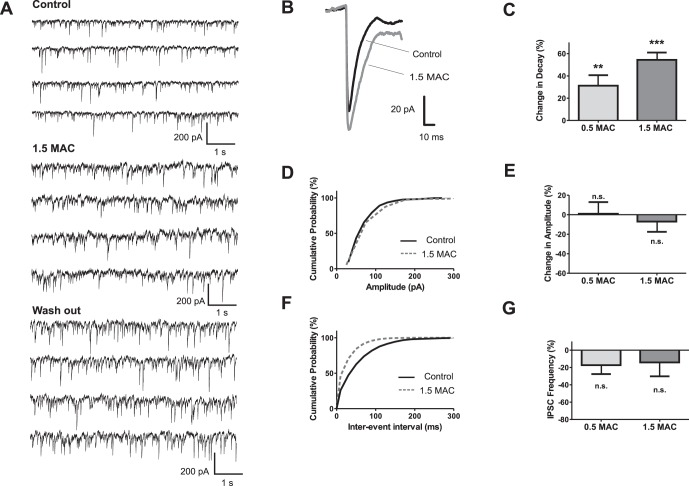
Glycinergic IPSC frequency remained stable at a high sevoflurane concentration. (**A**) Representative traces of glycine receptor-mediated IPSCs for control condition (*upper trace*), sevoflurane application (*middle trace*) and wash out (*lower trace*). Glycine IPSCs were isolated by adding CNQX (50 µM), AP5 (50 µM), and bicuculline (100 µM). (**B**) Overlay of the averaged IPSCs from the presented experiment in (*A*). (**C**) Summary of analyzed experiments revealed that sevoflurane significantly increased the IPSC decay time by 31.2±9.4% at 0.5 MAC (n = 6, p<0.01), and by 54.4±7.6% at 1.5 MAC (n = 12, p<0.001). Absolute decay time values were 8.02±0.52 ms for control (n = 18), 10.78±0.81 ms for 0.5 MAC (n = 6), and 11.82±0.87 ms for 1.5 MAC sevoflurane (n = 12). (**D**) Cumulative distribution of IPSC amplitudes from the representative experiment depicts that sevoflurane slightly shifted the population to higher amplitudes. (**E**) Pooled data showed that the mean of IPSC amplitudes was basically not changed under sevoflurane (change in amplitude 1.04±11.91% for 0.5 MAC, n = 6, and −7.01±10.46% for 1.5 MAC, n = 12). (**F**) In the presented experiment, sevoflurane reduced the inter-event intervals as shown by the cumulative distribution plot. (**G**) Data from all experiments revealed that sevoflurane did not significantly affect IPSC frequency at both concentrations (change in frequency −17.3±10.3% (n = 6) for 0.5 MAC, and −14.2±16.0% (n = 10) for 1.5 MAC). **p<0.01, and ***p<0.001 as indicated.

Similar to GABA_A_ receptor-mediated spontaneous IPSCs, the decay times of glycinergic spontaneous IPSC decay times were prolonged by sevoflurane concentration-dependently (**Fig. 4** **A, B, C**). In sharp contrast to GABAergic IPSCs, a significant effect of 1.5 MAC sevoflurane on the amplitudes of glycinergic IPSCs was not apparent (**Fig. 4** **D, E**). In addition, 1.5 MAC sevoflurane did not substantially affect the frequency of glycine receptor-mediated IPSCs (**Fig. 4** **F, G**).

### Anesthetic Modulation of Synaptic Efficacy

**Figure 5:**
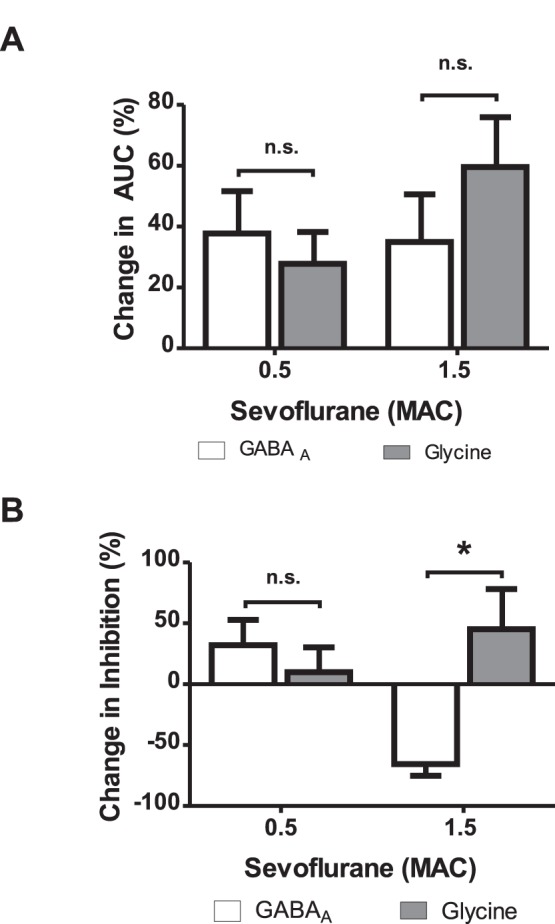
Sevoflurane altered the efficacy of GABAergic and glycinergic transmission. (**A**) Sevoflurane augmented the inhibitory charge transferred by an averaged synaptic event, which is described by the area under the curve (AUC). The AUC was calculated by multiplication of decay time and amplitude. The increase of the AUC did not differ between GABAergic and glycinergic transmission at low and high concentrations (0.5 MAC: GABA_A_ 37.75±13.9%, glycine 27.8±10.4%; 1.5 MAC: GABA_A_ 34.9±15.7%, glycine 59.6±16.4%, p>0.05 by ANOVA). (**B**) Synaptic inhibition transferred per time period was estimated by multiplication of the event frequency and the calculated AUC. At sub-anesthetic concentrations (0.5 MAC), synaptic inhibition was provided by both GABAergic and glycinergic systems, although the GABAergic transmission was more prominent (change in inhibition: GABA_A_ 32.2±21%, glycine 10±20%, ns. by ANOVA). Contrastingly, at high sevoflurane concentrations (1.5 MAC) the inhibition provided by GABA_A_ IPSCs was completely deteriorated (change in inhibition by −65.6±9.6%) while the glycinergic IPSC output was further augmented (change in inhibition by 45.2±33%, p<0.05 by ANOVA). *p<0.05 as indicated.

Next we explored how sevoflurane altered the efficacy of GABAergic and glycinergic synaptic transfer. An increase in synaptic efficacy is indicated by an increase in the amount of total charge transferred during the course of an averaged synaptic event (area under the curve). Synaptic efficacy can be enhanced either by an increase in amplitude or by prolongation of decay time of inhibitory events. At a sub-anesthetic concentration corresponding to 0.5 MAC equivalents, sevoflurane significantly increased the decay times of both, GABAergic and glycinergic IPSCs (**Fig. 2**, **Fig. 4**). Since IPSC amplitudes were not altered at this concentration and IPSC decay times were well fitted with single exponentials, the prolongation of decay times was translated into an increase in the total amount of charge transferred per IPSC (**Fig. 5** **A**). However, at 1.5 MAC sevoflurane equivalents, the decay time of GABAergic IPSCs was further prolonged but, on the other hand, the IPSC amplitudes were attenuated by roughly the same amount. These different actions of sevoflurane on the amplitude and decay time affected the charge transferred per IPSC in opposing ways and largely compensated each other. Thus, an increase of a sevoflurane concentration from 0.5 to 1.5 MAC equivalents was not associated with an increase in the efficacy of GABA-dependent inhibitory synapses in ventral horn interneurons (**Fig. 5** **A**, n.s. by ANOVA).

In contrast to GABA-mediated synaptic events, there was no reduction of glycinergic IPSC amplitudes detectable upon changing the concentration of sevoflurane from 0.5 to 1.5 MAC equivalents. But similar to GABAergic IPSCs, the decay time of glycinergic synaptic currents was prolonged. As a consequence, the efficacy of synapses operated by glycine further increased. However, statistical analysis did not reveal a difference between 0.5 and 1.5 MAC sevoflurane (**Fig. 5** **A**, n.s. by ANOVA).

### Sevoflurane Mediates Neuronal Inhibition Primarily Via Glycine Receptors at Concentrations Above 1 MAC

In a next step, we quantified the effects of sevoflurane on time-averaged synaptic transmission. This parameter depends on the efficacy of inhibitory synapses (quantified as the charge transferred per averaged IPSC) and on the frequency of synaptic events per time period. Time-averaged synaptic inhibition was estimated by the frequency of synaptic events multiplied by the charge transferred per averaged IPSC. The data displayed in **Fig. 5** **B** demonstrates that glycine-dependent synaptic inhibition of ventral horn interneurons slightly increases from 0.5 to 1.5 MAC equivalents, whereas GABA_A_ receptor-mediated inhibition strongly decreases (**Fig. 5** **B**). GABAergic inhibition differed significantly between 0.5 and 1.5 MAC sevoflurane concentrations (**Fig. 5** **B**, p<0.05, by ANOVA). Moreover, at 1.5 MAC sevoflurane glycine receptor-mediated inhibition was significantly higher in comparison to GABA_A_ receptor-mediated inhibition (**Fig. 5** **B**, p<0.05, by ANOVA).

### Different Efficacy of GABA_A_ and Glycine Receptor Antagonists in the Reversal of Neuronal Inhibition at Increasing Sevoflurane Concentrations

During voltage-clamp recordings glutamatergic transmission was blocked in order to isolate inhibitory postsynaptic currents. Therefore it seemed possible that the sevoflurane-induced changes in action potential-dependent GABAergic and glycinergic transmission as characterized above could only be observed under these specific experimental conditions.

**Figure 6:**
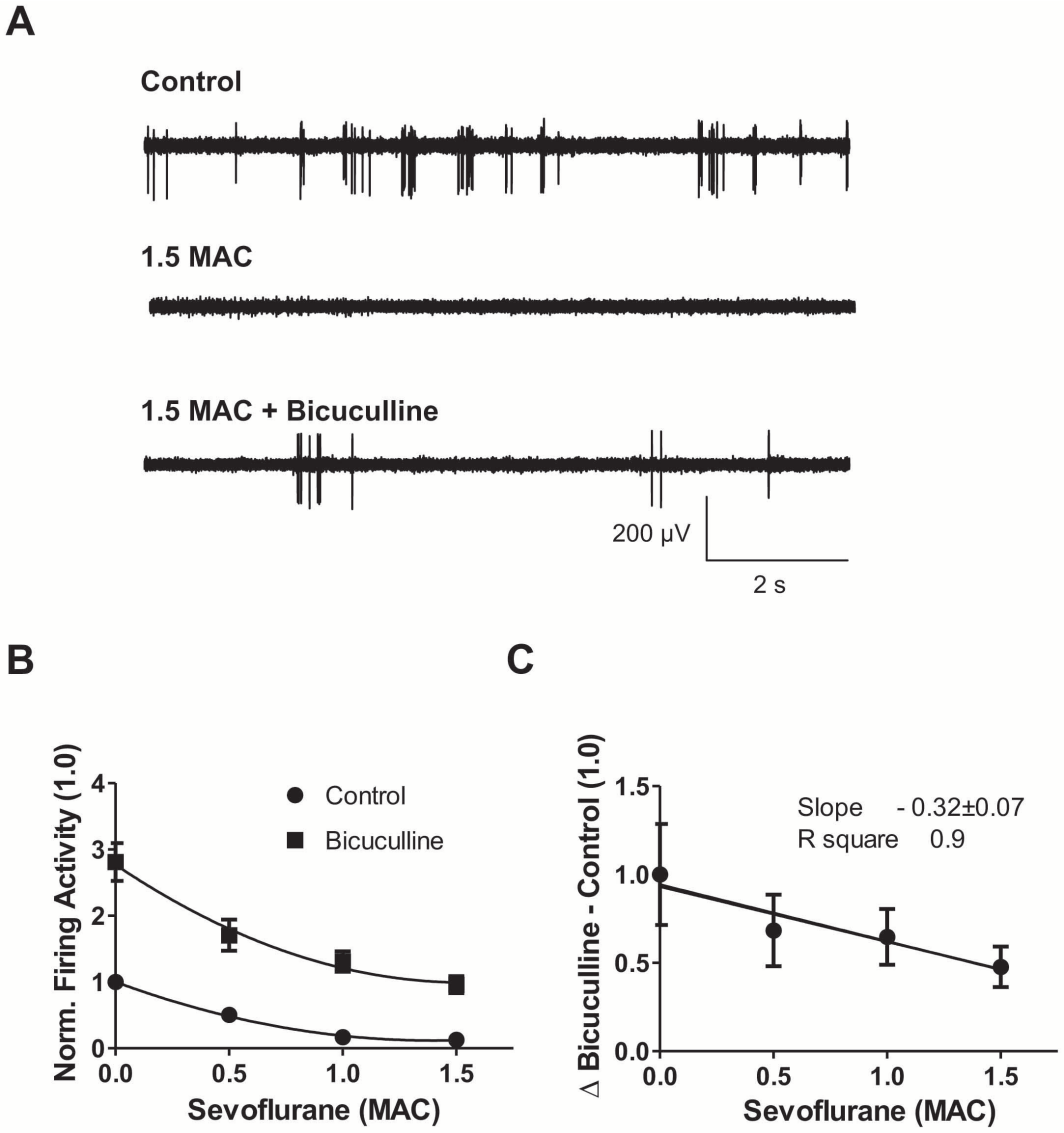
GABAergic transmission loses importance in action potential inhibition by increasing sevoflurane concentrations. (**A**) Representative traces of extracellular recordings before application of sevoflurane (*upper trace*), after application of 1.5 MAC sevoflurane (*middle trace*), and after application of 1.5 MAC sevoflurane and 100 µM bicuculline (*lower trace*). Complete depression of firing activity under sevoflurane and partial recovery under sevoflurane and bicuculline was observed. (**B**) Concentration-response curve of firing activity for control conditions and bicuculline (100 µM). For each sevoflurane concentration the mean±SEM is given and normalized to the respective control value. The curves were fitted with second order polynomial equations. Application of bicuculline increased the firing rate by about 2.8-fold. This portion decreased at higher sevoflurane concentrations. (**C**) The portion of GABA_A_ transmission was calculated by subtracting the control value from the respective bicuculline condition (68.3±20.3% of control for 0.5 MAC, n = 31; 64.6±15.7% for 1 MAC, n = 30 and 47.7±11.4% for 1.5 MAC, n = 30). Linear regression analysis depicts a decreasing role of the GABA_A_ receptor system at measured sevoflurane concentrations (slope −0.32±0.07, R square 0.9).

**Figure 7:**
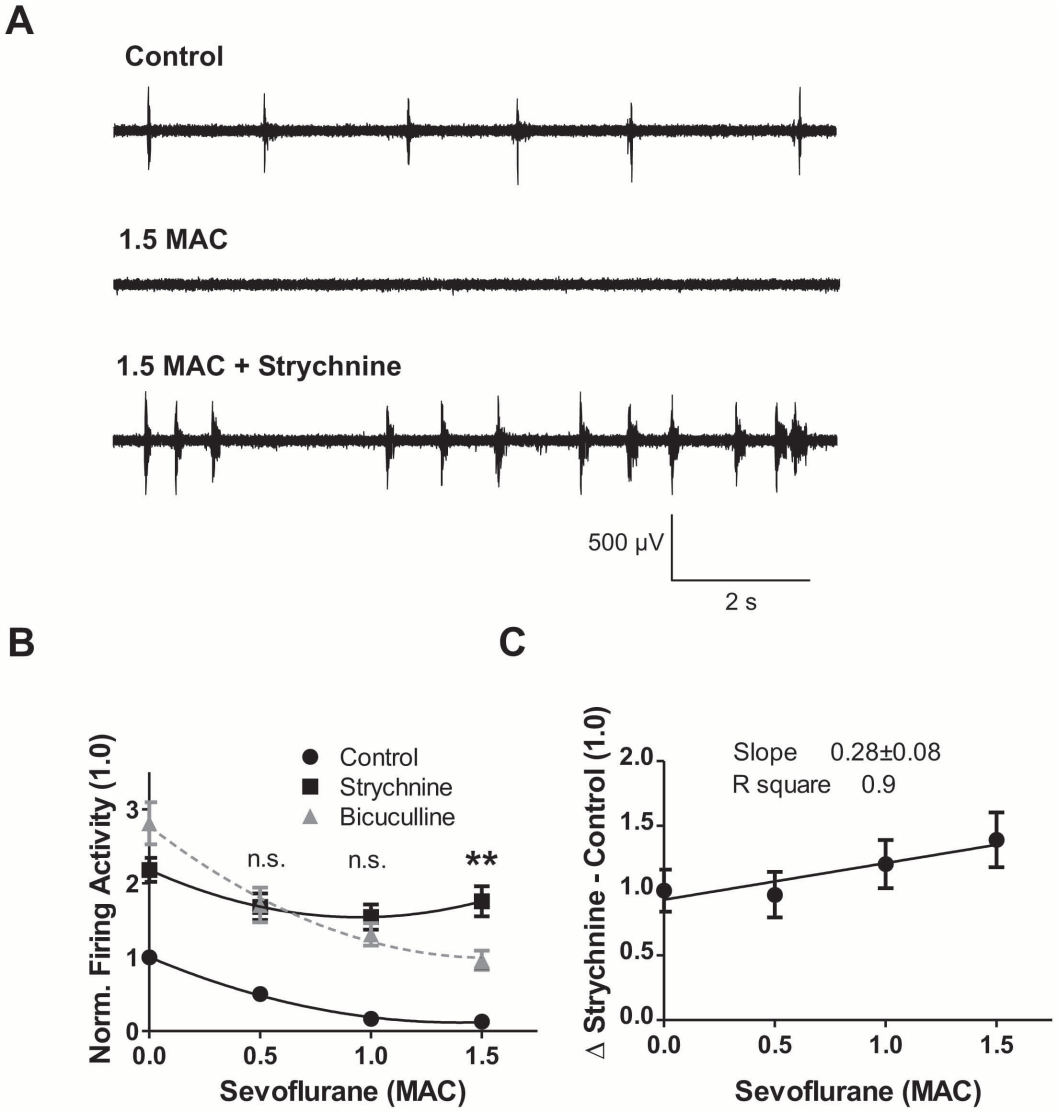
Augmenting role of glycinergic transmission at higher sevoflurane concentrations. (**A**) Original recordings showing the effect of 1.5 MAC sevoflurane on action potential firing in the ventral horn (*middle trace*; *upper trace* control). Additional application of the selective glycine receptor antagonist strychnine (1 µM) to the sevoflurane condition strikingly increased spontaneous network activity (*lower trace*). (**B**) The panel depicts the effect of strychnine on sevoflurane-induced firing suppression. In the presence of strychnine, a 2-fold increase in firing activity was observed. This portion was investigated under sevoflurane application. In contrast to GABA_A_ experiments (shown as *dotted gray line*), the application of the selective glycine antagonist strychnine almost overrode the sevoflurane effect. At 0.5 and 1.0 MAC equivalents, there was no difference detectable between the strychnine and bicuculline condition. At the high sevoflurane concentration (1.5 MAC) the impact of glycine was significantly larger compared with that from GABA (p<0.05, by ANOVA). Data was normalized to control conditions and presented as mean±SEM. Curve fittings were performed similarly as in *Fig. 6.* (**C**) Glycine contribution in sevoflurane-induced depression of action potential firing was calculated by subtracting the control value from the respective strychnine condition. Linear regression analysis demonstrates an increasing contribution of glycine receptor transmission to the effects of sevoflurane in a concentration-dependent manner (slope 0.28±0.08, R square 0.9; 96.8±17.6% of control for 0.5 MAC, n = 35; 120.6±18.6% for 1 MAC, n = 38; 139.3±21.2% for 1.5 MAC, n = 30).

To address this issue and to critically test the conclusions drawn from the results of voltage-clamp recordings, receptor antagonism experiments were conducted, leaving glutamatergic transmission intact. In these studies, extracellular multi-unit recordings of spontaneous action potentials were performed in organotypic spinal cord cultures for assessing the ability of sevoflurane to depress neuronal activity (**Fig. 6** **A**). The firing rate of ventral horn neurons was reduced by sevoflurane in a concentration-dependent manner (**Fig. 6** **B,** control group: 0.5 MAC: 50.4±4.0% of control firing activity (100%), n = 68; 1 MAC: 16.7±3.4% of control, n = 71; 1.5 MAC: 12.9±3.6% of control, n = 67). It can be assumed that GABA_A_ or glycine receptors are not involved in mediating sevoflurane effects, if the anesthetic depresses neuronal firing to the same extent in the presence of the corresponding receptor antagonist. Vice versa, the receptor is considered to be a major player in producing inhibition if sevoflurane is no longer effective in the presence of the respective antagonist. Taking this approach we quantified the effectiveness of sevoflurane in the presence and absence of the GABA_A_ receptor antagonist bicuculline and the glycine receptor antagonist strychnine, respectively (**Fig. 6** **A,** **Fig. 7** **A**). Application of the GABA_A_ receptor antagonist bicuculline partially reversed sevoflurane-induced depression of action potential firing (**Fig. 6** **A,** *middle and lower trace*). As anticipated from our voltage-clamp recordings, the potency of bicuculline to reverse the action of sevoflurane decreased at higher anesthetic concentrations (**Fig. 6** **B, C**).

In following experiments, the interactions between the selective glycine receptor antagonist strychnine and sevoflurane were investigated. Sevoflurane-induced inhibition of action potential activity was impressively reversed by strychnine (**Fig. 7** **A, B**). At anesthetic concentrations corresponding to 0.5 and 1.0 MAC, reversal of sevoflurane-induced depression of action potential firing, caused either by bicuculline or strychnine, occurred to a similar extent (**Fig. 7** **B**). However, this was not the case at an anesthetic concentration corresponding to 1.5 MAC (**Fig. 7** **B**, p<0.01 by ANOVA). In contrast to GABAergic inhibition, linear regression analysis uncovered an increasing impact of glycine receptors in sevoflurane-induced inhibition of action potential firing (**Fig. 7** **C,** slope 0.28±0.08, R square 0.9). In summary, these antagonism studies confirmed the major conclusion drawn from voltage-clamp recordings: At sevoflurane concentrations of 1.5 MAC glycine receptors are more important in mediating inhibition whereas GABA_A_ receptors lose their impact.

## Discussion

### Organotypic Cultures as Tool for Testing Sevoflurane Effects on Inhibitory Synaptic Transmission

Anesthetic effects on different neuronal substrates and their relation to immobility are not well understood. Recently, it could be shown that isoflurane caused a depression of spontaneous network activity in the spinal ventral horn. Modulation of the so-called central pattern generator activity was found to be essential in the disruption of motor output preceding immobility by isoflurane [16]. This inhibition was presumably independent of anesthetic effects on sensory or motor neurons [16]. Organotypic slices have been developed and successfully used to investigate central pattern generator-like activity *in vitro* [17]. The spontaneous rhythmic activity *in vitro* resembles the firing pattern observed during fictive locomotion [18], [19]. Another advantage of organotypic preparations is an easy access to individual interneurons since they flatten during culturing to a mono- or bilayer and allow a fast exchange of the perfusate [20]. Beyond investigations on central pattern generator-like activity, organotypic spinal slices have been successfully used for studying the effects of general anesthetics and neuroactive drugs [20], [21]. In the current study requirements on the model system included on the one hand easy access to single interneurons and on the other hand the existence of spontaneous neuronal network activity. Under these conditions sevoflurane actions on inhibitory postsynaptic currents were quantified in interneurons and related to network inhibition of the ventral horn.

### Multiple Actions of Sevoflurane on Inhibitory Synaptic Transmission in the Spinal Ventral Horn

In a pioneering study Zimmerman and coworkers showed that the volatile anesthetics isoflurane, enflurane and halothane prolonged the responses of GABA_A_ receptors to exogenously applied GABA [10]. Another study reported that the inhalational anesthetics sevoflurane, methoxyflurane, enflurane and isoflurane potentiated the responses of native and recombinant strychnine-sensitive glycine receptors at low glycine concentrations [22]. In both studies anesthetics were tested at concentrations corresponding to 1 MAC. Therefore it was hypothesized that both types of receptors might contribute to the immobilizing properties of volatile anesthetics. This idea was further backed by the landmark paper of Mihic and coworkers, who identified a common binding site for volatile anesthetics and ethanol on GABA_A_ and glycine receptors [9]. In summary, these observations strongly suggested a large degree of overlap between the effects of inhalational anesthetics on GABA_A_ and glycine receptors.

However, there is strong evidence that volatile anesthetics both enhance and inhibit the function of GABA_A_ receptors [23], [24]. At intact GABAergic synapses, the potentiating action of volatile anesthetics manifests as an increase in the decay time of synaptic currents, whereas inhibition is evident as a decrease in the amplitude of the synaptic events [25]–[27]. Banks and Pearce demonstrated that the blocking effect of enflurane and isoflurane was of postsynaptic origin [26]. Furthermore, the concentration-dependence of the prolonging and blocking effect dissociated as the latter effect required higher concentrations [25], [26]. The authors proposed that distinct mechanisms might underlie the two actions.

Due to the dual actions of volatile anesthetics on GABA_A_ receptors, the charge transferred during an averaged IPSC is not monotonically rising, when the concentration of the volatile anesthetic is increased [26]. For miniature IPSCs recorded from hippocampal pyramidal cells, the total charge transferred during IPSCs peaked at an enflurane concentration corresponding to 1 MAC equivalents. The same result was obtained for both, GABA_A_-mediated spontaneous IPSCs as well as miniature IPSCs from cerebellar Purkinje cells [25]. Thus, at concentrations smaller than 1 MAC the prolonging effect of enflurane was dominant, whereas at concentrations higher than 1 MAC the blocking effect over-compensated the prolonging effect. Nishikawa and MacIver investigated the effects of volatile anesthetics on spontaneous and evoked GABAergic IPSCs in acute hippocampal brain slices [28]. They found dual effects for halothane, enflurane, isoflurane, and sevoflurane with different magnitudes for the changes in prolongation of IPSCs as well as for the reduction of amplitudes [28]. In contrast to the reduction of IPSC events observed in the current study, they demonstrated a two- to threefold increase in IPSC frequencies causing a serious enhancement of GABA_A_ receptor-mediated synaptic inhibition in hippocampal interneurons by volatile anesthetics in an agent-specific manner [28]. The resulting hypothesis that volatile anesthetics modulate GABAergic currents brain region-dependent is strengthened by a study recently published by Oose and coworkers [29]. This work reported a sevoflurane-induced depression of inhibitory charge transfer in the striatum, while an enhancement of GABAergic current occurred in hippocampal CA1 pyramidal neurons.

In the ventral horn of cultured spinal tissue slices, action potential-dependent synaptic inhibition mediated via GABA_A_ and glycine receptors is much more powerful as compared to action potential-independent neurotransmitter release (miniature IPSCs). Therefore we focused on the former type of synaptic events. The key finding is that the time-averaged synaptic inhibition of ventral horn interneurons mediated by GABA_A_ receptors is strongly reduced at 1.5 MAC sevoflurane equivalents, whereas glycine receptor-mediated inhibition of the same neurons further increased. Depression of GABAergic transmission is, to a smaller fraction, due to a decrease in the IPSC amplitude and to a larger extent to a dramatic decrease in IPSC frequency. The latter effect possibly involves a decreased action potential firing rate of GABA-releasing interneurons. This result is indeed consistent with our previous report on the effects of etomidate on ventral horn interneurons [30]. Similar to sevoflurane, etomidate strongly reduced the frequency of action potential-dependent GABAergic synaptic events. Interestingly, this effect was abolished by the beta3 (N265M) mutation, proving that action potential-dependent GABA release in the ventral horn is controlled by beta3 subunit containing GABA_A_ receptors [30]. It is reasonable to assume that this GABA_A_ receptor subtype is also sensitive to sevoflurane.

In contrast to GABA_A_ receptor-dependent inhibition, a high sevoflurane concentration was not accompanied by pronounced changes in glycine receptor-mediated IPSC amplitudes or frequency of the events, demonstrating that the synaptic transmission at GABAergic and glycinergic synapses is differently modified by sevoflurane. This conclusion is also supported by the observation that enflurane, at a concentration equivalent to 1 MAC, does not diminish the amplitude of glycinergic miniature IPSCs, although at the same concentration enflurane strongly attenuates the amplitudes of GABA-dependent miniature IPSCs [31]. In summary, there is now ample evidence that volatile anesthetic-induced potentiation of GABAergic synaptic transmission in the spinal ventral horn is limited by a blocking effect on GABA_A_ receptors and a decrease in GABA release. Both actions get effective at a concentration corresponding to 1 MAC and above. These findings are in good accordance with the conclusion that the effects of volatile anesthetics on GABA_A_ receptors are not of major importance in causing immobility [12].

### Limitations of the Study

At clinically relevant concentrations, volatile agents provide immobility by acting on a concert of various ligand-gated and voltage-gated ion channels [32]–[36]. Additionally, recent work has focused on background potassium channels (TASK) in providing immobility by inhaled anesthetics [15], [37]. TASK channel knock-out mice were less sensitive to volatile anesthetics requiring higher halothane or isoflurane concentrations to render mice unresponsive to tail pinching [15], [37]. High levels of mRNA expression of TASK-1 and -3 were observed in ventral horn motoneurons [38] and presynaptic mechanisms linked to potassium channels could also affect synaptic transmission in the spinal cord. In our study, more than 99% of spontaneous GABAergic synaptic events were action potential triggered (**Fig. 3**). This finding highlights the importance of the action potential-generating machinery including sodium channels in the ventral horn. Since volatile anesthetics inhibit neuronal voltage-gated sodium channels at clinical concentrations, these currents might interplay in providing immobility [32], [39]. Further, presynaptic alterations of locomotor interneurons due to direct or indirect actions on calcium may influence the inhibitory transmission [40]. In summary, these findings strongly suggest that anesthetic actions on GABA_A_ and glycine receptors, which were studied here, are not the sole, but important mechanisms by which sevoflurane acts in the spinal ventral horn.

## Materials and Methods

### Spinal Slice Cultures

All procedures were performed in accordance with institutional and federal guidelines, including the German law on animal experimentation, and were approved by the animal care committee (Eberhard-Karls-University, Tübingen, Germany) and the Regierungspräsidium Tübingen. Spinal slice cultures were prepared as first described by Braschler and coworkers [41]. Briefly, pregnant C57BL6 mice (day E 14–15) were anesthetized and decapitated [13]. Embryos were placed into ice-cold Gey’s balanced salt solution consisting of 1.5 mM CaCl_2_, 5 mM KCl, 0.22 mM KH_2_PO_4_, 11 mM MgCl_2_, 0.3 mM MgSO_4_, 137 mM NaCl, 0.7 mM NaHCO_3_, and 33 mM glucose (all from Sigma, Taufkirchen, Germany). After decapitation of the embryos, spinal columns were transversely cut into 300 µm thick slices using a microslicer (NVSLM1, World Precision Instruments, Sarasota, FL, USA). Slices were transferred to coverslips and embedded in heparin-treated chicken plasma (Sigma). In a second step, thrombin solution (Sigma) was added for allowing clot formation. The coverslips were put into plastic tubes with 0.75 mL nutrient fluid containing 10 nM neuronal growth factor (Sigma) and incubated in 95% oxygen/5% carbon dioxide at 36.0°C. Nutrient fluid (100 mL) consisted of 25 mL horse serum (Invitrogen, Karlsruhe, Germany), 25 mL Hanks’ balanced salt solution (Sigma), 50 mL basal medium Eagle (Sigma), 1 mL 50% glucose, and 0.5 mL L-glutamin (200 mM). The roller tube technique was used to culture the tissue [42]. After 1 day in culture, antimitotics (10 µM 5-fluoro-2-deoxyuridine, 10 µM cytosine-β-D-arabino-furanoside, 10 µM uridine, all from Sigma) were added to reduce proliferation of glial cells. Slices were typically used after 21.7±6.3 (mean±sd) days *in vitro* for extracellular recordings (n = 201), and after 15.5±4.1 (mean±sd) days *in vitro* for whole-cell voltage-clamp recordings (n = 34).

### Extracellular Recordings

Spontaneous action potential activity was recorded as reported previously [13]. In brief, slices were perfused with carbogenated artificial cerebrospinal fluid (ACSF) consisting of (mM) 120 NaCl, 3.5 KCl, 1.13 NaH_2_PO_4_, 1 MgCl_2_, 26 NaHCO_3_, 1.2 CaCl_2_, and 11 D-glucose. Glass electrodes with a resistance of approximately 2–5 MΩ were filled with ACSF and were introduced into the ventral horn area until extracellular spikes (usually exceeding 100 µV in amplitude) were visible and single-unit or multiunit activity could be clearly identified. The noise (peak-to-peak) amplitude was usually approximately 50 µV. All experiments were performed at 34°–35°C. Signals were bandpass-filtered (passband 200–5000 Hz) and digitized at 10 kHz via a Digidata 1200 interface and Axoscope 9.0 software (Molecular Devices, Sunnyvale, CA). At stable control conditions the inhibitory effect of sevoflurane on action potential firing was quantified at different concentrations in the ventral horn network. For estimating the impact of glycinergic transmission, the selective glycine receptor antagonist strychnine (1 µM) was added to the respective sevoflurane concentration. The action potential firing rate was compared with the value obtained from the strychnine condition. Reaching control conditions assumed a 100% contribution of the particularly blocked receptor in mediating inhibitory sevoflurane actions, whereas an unchanged firing rate implied no involvement of the blocked receptor in sevoflurane action. To assess the contribution of GABA_A_-mediated network suppression by sevoflurane, 100 µM bicuculline, a selective GABA_A_ receptor antagonist, was washed in (all from Sigma, Germany). Previous experiments demonstrated that a lower bicuculline concentration (20 µM) did not completely block GABA_A_ receptor-induced conductance [43].

### Whole-cell voltage-clamp Recordings

Whole-cell voltage-clamp recordings were performed on visually identified ventral horn interneurons at room temperature (20–24°C) as described previously [30]. In the recording chamber, cultures were continuously perfused with ACSF as specified above. 6-cyano-7-nitroquinoxaline-2.3-dione (CNQX; 50 µM) and D-L-2-amino-5-phosphonopentanoic acid (AP5, 50 µM) was added to suppress glutamatergic transmission. For isolating GABA_A_ inhibitory postsynaptic currents (IPSCs) 1 µM strychnine was added and for glycine-mediated IPSC recordings 100 µM bicuculline (all from Sigma). Signals were acquired with a Multiclamp 700B patch-clamp amplifier (Molecular Devices, Foster City, CA) equipped with a CV-7B headstage, low-pass-filtered at 2.2 kHz, and digitized at 10 kHz via a Digidata 1440 A interface and Clampex 10.1 (Molecular Devices, Foster City, CA). Patch pipettes were pulled from thin-wall borosilicate capillaries (World Precision Instruments, Sarasota, FL, USA) leading to initial pipette resistances between 3 and 5 MΩ. The pipette solution contained (mM) 121 CsCl, 24 CsOH, 10 HEPES, 5 EGTA, 1 MgCl_2_, and 2 ATP, adjusted to pH 7.2 with 1 M HCl (all from Sigma). Cells were clamped at −70 mV voltage potential.

### Preparation and Application of the Volatile Anesthetic Sevoflurane

As previously reported, test solutions containing sevoflurane were obtained by dissolving the liquid form of the anesthetic in ACSF, which was equilibrated with 95% oxygen and 5% carbon dioxide [13]. As MAC of sevoflurane has not been defined in mice, rat MAC values (minimal alveolar concentration; 1 MAC suppresses movement in response to noxious stimulation in 50% of subjects) were used throughout the experiments [44], [45]. However, a previous study showed that the MAC requirements of halothane, isoflurane and enflurane were less than 10% higher in rats compared with those in mice [46]. According to Franks and Lieb, 1 MAC corresponds to an aqueous concentration of 0.35 mM sevoflurane [45]. Regarding potential variations of MAC values at different temperatures Franks and Lieb could show that aqueous halothane concentrations were in the narrow range of 0.18–0.23 mM over the broad temperature range 20–37°C in experiments *in vitro* [45]. Therefore, they concluded that a MAC value specified for 37°C should also be a reasonable approximation for the use at room temperature [45].

For measuring the actual sevoflurane concentration in ACSF, samples containing nominal 2 MAC (25 mL ACSF) were stored in gas-tight glass syringes (diluted with room air to a volume of 50 mL therewith to a final sevoflurane concentration of 1 MAC) overnight at 37°C. After incubation, the gas-phase concentration was measured using a gas monitor (Oxyanga, Heinen & Löwenstein, Bad Ems, Germany). The detected sevoflurane concentration in the gas-phase was 0.766±0.033 MAC (n = 3), thereby about 20% lower than the nominal 1 MAC. This difference can be explained by the dead space volume of the analyzing monitor.

Sevoflurane was administered via bath perfusion using a gas-tight syringe pump system (ZAK Medicine Technique, Marktheidenfeld, Germany), which was connected to the recording chamber via Teflon tubing (Lee, Frankfurt, Germany). Measurements by gas chromatography revealed that losses of volatile agents from the perfusion system are negligible, when similar precautions are performed [22], [47]. The flow rate was set at 1 mL/min. To ensure steady state conditions, recordings during anesthetic treatment were conducted 10–15 min after starting wash in [13]. The calibration of the recording system was performed as previously reported [13].

### Data Analysis

Data were analyzed with in-house software written in OriginPro version 7 (OriginLab Corp, Northampton, MA) and MATLAB version 7.7 (The MathWorks Inc., Natick, MA). IPSC events were detected by setting a threshold criterion well above the baseline. To ensure that sevoflurane-induced reduction of IPSC frequency was not a result of a deteriorated detection due to impaired amplitudes, the fraction of IPSC which might potentially fall below threshold level was calculated. As amplitudes of GABA_A_ receptor-mediated IPSCs were decreased by 30% at 1.5 MAC sevoflurane, the threshold criterion (*i.e.* 50 pA) was divided by 0.7 (subtracting 30% = 70% of control) to estimate the critical IPSC amplitude (*i.e.* 50 pA divided by 0.7 = 71 pA). Under control conditions the determined IPSC fraction less or equal of the critical IPSC amplitude was 18.7±1.8% (n = 8). IPSC current decays were well fitted with a mono-exponential function. Graphs were generated by using GraphPad Prism 5. Comparative statistics were performed by using a two-tailed Student's t-test for paired data or one-way ANOVA followed by a Newman-Keuls multiple comparison post hoc test when indicated (*p<0.05, **p<0.01, ***p<0.001). p≥0.05 was defined as non significant (n.s.). Data analysis of extracellular recordings was performed as described previously [13]. Action potentials were detected by setting a threshold well above baseline noise. The mean firing rate obtained from single or multiunit activity was defined as the number of detected action potentials divided by the recording period of 180 s. The natural firing mode of spinal ventral horn neurons in culture consisted of bursts and silent periods. Inhibitory effect of sevoflurane on network firing activity was fitted best with second order polynomial equation as proposed by Antkowiak and Heck [25]. Data was fitted with a linear regression line when indicated. All data is presented as mean±S.E.M. Number of analyzed experiments (n) is given.
